# Tectorigenin Alleviates Inflammation, Apoptosis, and Ossification in Rat Tendon-Derived Stem Cells *via* Modulating NF-Kappa B and MAPK Pathways

**DOI:** 10.3389/fcell.2020.568894

**Published:** 2020-10-22

**Authors:** Safwat Adel Abdo Moqbel, Kai Xu, Zhonggai Chen, Langhai Xu, Yuezhe He, Zhipeng Wu, Chiyuan Ma, Jisheng Ran, Lidong Wu, Yan Xiong

**Affiliations:** ^1^Department of Orthopedic Surgery, The Second Affiliated Hospital, School of Medicine, Zhejiang University, Hangzhou, China; ^2^Department of Orthopaedic Surgery, The Second Affiliated Hospital and Yuying Children’s Hospital of Wenzhou Medical University, Wenzhou, China; ^3^Department of Orthopaedics, The First Affiliated Hospital of Zhejiang Chinese Medical University, Hangzhou, China

**Keywords:** TDSCs, tectorigenin, TNF-α, inflammation, apoptosis, ossification, MAPK, nuclear factor-kappa B pathway

## Abstract

Tendinopathy is a common musculoskeletal disorder that mainly affects athletes and people of older age. Tumor necrosis factor-α (TNF-α) plays an important role in initiating tendinopathy. Tectorigenin, an extract component of *Belam-canda Chinesis*, possesses anti-inflammatory and anti-apoptosis activity. The present study was established to investigate the role of tectorigenin against the pathogenetic effects of TNF-α on tendon-derived stem cells (TDSCs) *in vivo* and *in vitro*. The findings indicated that TNF-α is able to induce TDSC inflammation, apoptosis, and ossification, as well as activate nuclear factor-kappa B and mitogen-activated protein kinase (MAPK). Furthermore, the results confirmed that tectorigenin is able to inhibit the TNF-α-induced inflammation, apoptosis, and ossification. Tectorigenin treatment decreases activation of NF-kappa B and MAPK signaling in TDSCs. Tectorigenin ameliorates tendinopathy in the *in vivo* rat model. Thus, these data reveal that tectorigenin can serve as a potential treatment for tendinopathy.

## Introduction

Tendinopathy is a chronic disorder characterized by swelling, pain, ossification, and dysfunction of the tendon. Previous studies have shown that several factors can contribute to tendinopathy, such as tendon overload, injury, aging, and genetic conditions (Almekinders and Temple, 1998). Tendon-derived stem cells (TDSCs), which are extracted from tendon tissues, possess self-renewal and tendon-like tissue regeneration capabilities (Bi et al., 2007). TDSCs play a central role in tendon regeneration and healing as well as controlling tendon homeostasis (Ni et al., 2012). A change in their microenvironment leads to the dysfunction of TDSCs, resulting in degradation of tendon matrix and ultimately tendinopathy (Shi et al., 2019).

TNF-α is a cytokine associated with tendon inflammation, degeneration, and apoptosis, and it contributes to the suppression of proliferation of TDSCs (Hosaka et al., 2005; Han et al., 2017). TNF-α is able to induce the production of matrix degradation enzymes (Machner et al., 2003), which are associated with the inhibition of ECM synthesis (John et al., 2010). TNF-α is released from tendon tissue during injury and malfunction (Millar et al., 2009; Morita et al., 2017). TNF-α was found to play an important role in tendon degeneration and was expressed in inflamed and scarred tendon (Schulze-Tanzil et al., 2011). TNF-α is well-known to play a role in initiating various signaling pathways, such as mitogen-activated protein kinase (MAPK) and nuclear factor kappa B (NF-κB pathways) (Akiyama et al., 2004). In addition, MAPK and NF-κB are known to govern several processes, such as inflammation, MMP secretion, apoptosis, and ossification in tendinopathy. The inhibition of TNF-α and targeting of MAPK and NF-κB offers potential in the treatment of tendinopathy. Previous studies have suggested and recognized Achilles tendon injury or Achilles tenotomy as a reproducible trauma-induced tendinopathy and heterotopic ossification model (Kaleağasioğlu et al., 2017; Lin et al., 2018). Therefore, in our study we established the rat tendinopathy model using tenotomy.

Tectorigenin (C16H12O6, Service number: 548-77-6; molecular weight, 300.26) a component of *Belamcanda chinensis*, has been used in several fields for its modulating effect against inflammation, oxidation, and osteoclastogenesis (Wang et al., 2013; Ma et al., 2018). Furthermore, tectorigenin is able to inhibit the MAPK and NF-κB signaling pathways (Lim et al., 2018; Ma et al., 2018). In the present study, we examined the role of tectorigenin on the inflammation, apoptosis, and ossification of TDSCs through the targeting of MAPK and NF-κB *in vitro*, in addition to its effect in tendinopathy in the rat model.

## Materials and Methods

### Materials

Tectorigenin of purity higher than 98% was purchased from Biotech (Shanghai, China). Fetal bovine serum (FBS), minimum essential medium with alpha modification (α-MEM), streptomycin, and penicillin were purchased from Gibco, United States. TNF-α was obtained from R&D Systems, United Kingdom. DMSO, bovine serum albumin (BSA), and collagenase I were all obtained from Sigma-Aldrich, St. Louis, MO, United States. Radioimmunoprecipitation assay (RIPA) buffer and bicinchoninic acid assay were obtained from Beyotime, Shanghai, China.

### Isolation and Culture of TDSCs

Tendon-derived stem cells were isolated and cultured as previously described (Xu et al., 2020). In brief, S-D rats (male, 3-week old, 140 ± 20 g) were used to isolate the Achilles tendons. For isolation of TDSCs from tendinopathic rats, five rats received full Achilles tendon transection. After 7 days, the rats were killed to isolate the tendinopathic cells. Under sterile conditions, the tendons were cut into 1.1 mm^3^ particles, and the tissues were incubated with 0.1% collagenase type I at 37°C for 2–3 h on a horizontal shaker. The cells were collected as P0 and incubated with α-MEM (with 10% FBS and 100 units/ml streptomycin + 100 units/ml penicillin) at 37°C with 5% CO_2_, and the culture medium was replaced every 2–3 days. TDSCs from tendinopathic rats were seeded and divided into two tendinopathy and tectorigenin treatment groups. The medium was replaced every 2 days; cells in the tectorigenin treatment group received a medium containing an equal amount of 100 μM of tectorigenin. After the density of cells in the culture flask reached more than 80%, the cells were harvested for a further study. Normal TDSCs at P3 were used in this experiment.

### Identification of TDSC and Multipotency Assay

Tendon-derived stem cells were stained with fluorescent primary antibody in PBS for 40 min, and then washed three times. Flow cytometry was used to detect the surface markers. The following antibodies were used: FITC antirat (CD29 and CD44) and PE antirat (CD59 and CD90) BioLegend. To analyze the multipotency of TDSCs, cells were cultured and incubated with specific media. Osteogenic induction medium (Cyagen Biosciences) was used for 14 days, followed by Alizarin Red staining to visualize the differentiation of TDSCs into osteoblasts. Chondrogenesis was induced with chondrogenic differentiation medium (Cyagen Biosciences) for 21 days, after which Safranin O staining was used to visualize chondrogenic differentiation. Similarly, after incubation with adipogenic induction and maintenance media (Cyagen Biosciences) for 14 days, Oil Red staining was used to visualize the adipogenic differentiation of TDSCs. qRT-PCR was conducted to evaluate the markers of tenogenesis in TDSCs and other mesenchymal stem cells. The mRNA levels of collagen type I, scleraxis, tenomodulin, and mohawk were evaluated.

### TDSC Viability Assay

To measure the toxicity of tectorigenin and TNF-α on TDSC, CCK-8 assay (Dojindo Molecular, Tech., Japan) was used in accordance with the manufacturer’s instructions. The cells were seeded into 96-well plates (5 × 10^3^/well), and then treated with various concentrations of tectorigenin or TNF-α for 24 h. The cells were then incubated with fresh media (containing 10% CCK-8 solution) for 3 h at 37°C; thereafter, the optical density was read. This experiment was repeated three times.

### Apoptosis Assay

Annexin-V-FITC/Propidium Iodide (PI) Apoptosis Detection Kit (Keygen Biotech) was used to detect the apoptotic cells. A mount of 10^6^ TDSCs was double stained using the apoptosis detection kit and analyzed using flow cytometry.

### RNA Extraction and qRT-PCR

Six-well plates were used to seed a tertiary culture (passage 3) of TDSCs in α-MEM. When the growth density of the cells was greater than or equal to 75%, tectorigenin at 50 and 100 μM was used to treat the cells for 1 h in the absence and presence of TNF-α for 24 h individually. After the media was removed and the cells were washed with PBS, TRIzol reagent (Invitrogen, Carlsbad, CA, United States) was used to extract total RNA on ice. Using DeNovix at 260 nm (A260)/A280 absorbance ratio total RNA was quantified. PrimeScript RT Master Mix (Takara) was used to synthesize cDNA. cDNA was synthesized at 37°C for 15 min and then at 85°C for 5 min. According to protocol, SYBR Green qPCR (Applied Biosystems) and StepO-nePlus Real-Time PCR systems were used to conduct qRT-PCR. The primers are listed in Table 1. 18S was used as the control. Expression levels were quantified using the 2^–ΔΔ*CT*^ method.

**TABLE 1:** Primer sequences used in this study.

Gene	Forward	Reverse
MMP-3	CAGGCATTGGCACAAAGGTG	GTGGGTCACTTTCCCTGCAT
MMP-9	GCAAACCCTGCGTATTTCCAT	GATAACCATCCGAGCGACCTTT
MMP-13	GCAAACCCTGCGTATTTCCAT	GATAACCATCCGAGCGACCTTT
COX-2	GAGAGATGTATCCTCCCACAGTCA	GACCAGGCACCAGACCAAAG
iNOS	CCTACGAGGCGAAGAAGGACAG	CAGTTTGAGAGAGGAGGCTCCG
Col1	GAGAGCATGACCGATGGATT	CCTTCTTGAGGTTGCCACTC
Runx-2	ACTTCCTGTGCTCGGTGCT	GACGGTTATGGTCAAGGTGAA
Scx	AACACGGCCTTCACTGCGCTG	CAGTAGCACGTTGCCCAGGTG
Mkx	TTTACAAGCACCGTGACAACCC	ACAGTGTTCTTCAGCCGTCGTC
Tnmd	TGGGGGAGCAAACACTTCTG	TCTTCTTCTCGCCATTGCTGT
IL-6	AGCGATGATGCACTGTCAGA	GGAACTCCAGAAGACCAGAGC
IL-10	TTCCATCCGGGGTGACAATAA	TTCTGGGCCATGGTTCTCTGC
18S	CCTGAGAAACGGCTACCACA	ACCAGACTTGCCCTCCAATG

### Protein Isolation and Western Blot

Tendon-derived stem cells were cultured in four 25 cm^2^ flasks and treaded with tectorigenin. After collecting the cells, 100 μl RIPA buffer (containing 1% phenylmethylsulfonyl fluride + 0.1% phosphorylated proteinase inhibitor) was used to extract total protein for 50 min. BCA assay was used to quantify the proteins which were then denatured at 95–100°C for 5 min. The adjusted proteins were set in SDS-PAGE with a 10 or 15% gel and then transferred to a membrane. The membrane was blocked with BSA (5–10%) for 1–2 h and then washed three times for 10 min. After, the membranes were incubated with primary antibodies for 12 h at 4°C. Subsequently, the membranes were washed and incubated with secondary antibodies for 1 h. After removing and washing the secondary antibodies, the membranes were read using an ECL kit (Immobilon; cat. no. WBKLS005; KGaA). Bio-Rad ChemiDoc system was used to quantify the protein bands.

### Radiological Assessment

Calcifications of Achilles tendons of the rats were evaluated using X-ray machine. Lateral X-ray images of the legs of the rats were generated at 60 kV with a radiation intensity of 500 mAs (200 M a).

### ALP Staining

Tendon-derived stem cells were seeded into 12-well plates with osteogenic induction medium with or without tectorigenin for 7 days. After washing with PBS, 4% paraformaldehyde was used to fix the cells for 30 min. ALP Color Development Kit (Beyotime Biotechnology, Shanghai, China) was used according to the protocol and then viewed under a microscope.

### Alizarin Red Staining

The cells were incubated in osteogenic differentiation media for 14 days and were washed with PBS. The cells were fixed with 4% paraformaldehyde for 20 min, and subsequently stained with 0.1% solution of Alizarin Red (Cyagen Biosciences) for 15 min, to identify mineral depositions and calcium-containing osteocytes.

### Senescence Assessment

Cells were cultured in 12-well plates with α-MEM medium and then treated with tectorigenin for 1 h followed by TNF-α for 24 h. According to the manufacture’s protocol, the β-galactosidase Activity Assay (Beyotime) was used to stain the cells for 24 h and the images were captured with microscope.

### Immunofluorescence

The TDSCs were prepared and then treated with tectorigenin for 1 h. The cells were then incubated with 10 ng/ml followed by TNF-α for 30 min. Subsequently, the cells were washed with PBS and then fixed with methanol for 20 min; 0.5% (*v*/*v*) Triton X-100 was used to permeabilize the cells and then blocked with 5% BSA for 1 h. The cells were then incubated with primary antibody against P65 at 4°C for more than 12 h, followed by incubation with fluorescein isothiocynate-conjugated secondary antibody for 2 h. After washing with PBS, the nucleuses were stained with DAPI for 7 min, and then analyzed using fluorescence microscopy.

### Animal Experiment

Eighteen Sprague-Dawley rats (male; 200 ± 10 g; 6 weeks old) were used in this experiment. The rats were divided into normal, tectorigenin-treated, and tendinopathy model groups. The tectorigenin-treated and tendinopathy model rats underwent Achilles tenotomy (Jiang et al., 2018). After anesthesia, the skin was shaved and sterilized. Subsequently, the skin and the paratenon were incised, and the fascicles of the Achilles tendon received full transection (5 mm proximal to the calcaneal insertion) perpendicular to the collagen fibers. After 1 week, the rats in the tectorigenin-treated group each received 100 μM tectorigenin once a week at the area between the Achilles tendon and skin for 8 weeks Supplementary Figure 2, and the limbs of the rats were harvested thereafter. All animal experiment studies were controlled and approved by the Ethics Committee of the Second Affiliated Hospital, School of Medicine, Zhejiang University, Hangzhou, China.

### Histological Analysis and Immunohistochemical Staining

The tendon samples were cut into 4 μM sections and then stained with HE, terminal deoxynucleotidyl transferase dUTP nick end labeling (TUNEL), and modified Masson staining according to their protocol. Immunohistochemical staining was conducted to evaluate tendinopathy in the tendon sections. The tendon sections were prepared and then subjected to antibodies against MMP-3 and MMP-13.

### Statistical Analysis

All data were represented as mean ± SD, and one-way ANOVA with a subsequent *post hoc* Tukey method was performed for multiple comparisons. A *P-*value of less than 0.05 was considered significant.

## Results

### Identification of TDSCs

**FIGURE 1:**
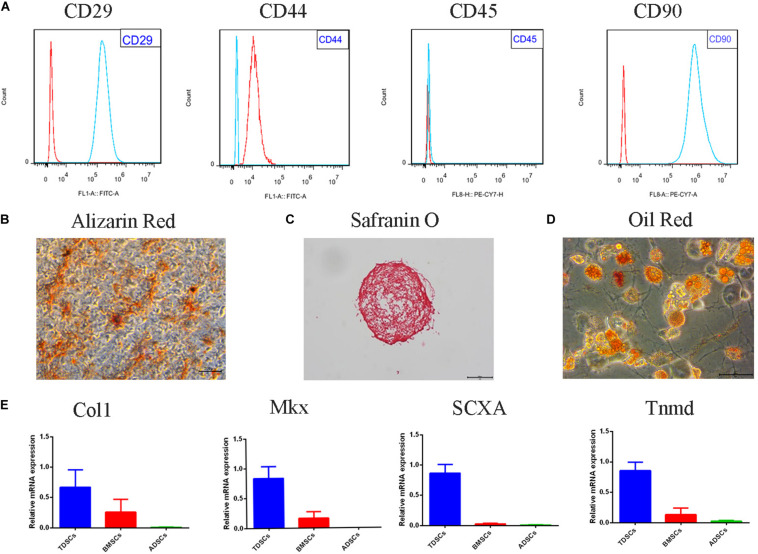
Tendon-derived stem cell (TDSC) identification and the multipotency properties. The identification of the stem status of TDSCs was performed by the analysis of surface markers. **(A)** Representative flow cytometric data of TDSCs incubated with CD29, CD44, CD45, and CD90 (red for control and blue for fluorescent antibody). **(B)** Alizarin Red stain (osteogenesis) (Scale bar = 500 μm). **(C)** Safranin O stain (chondrogenesis) (Scale bar = 500 μm). **(D)** Oil Red stain (adipogenesis) (Scale bar = 50 μm). **(E)** qRT-PCR of tenogenesis markers of TDSCs. Col1, collagen type I; SCX, scleraxis; Tnmd, tenomodulin; mkx, mohawk.

The TDSC surface marker analysis was conducted to identify the stem status of the cells. The findings confirmed that clonogenic cells express high levels of stem cell markers CD29, CD90, and CD44. The leukocyte marker CD45 showed undetectable levels (Figure 1A). In order to verify characteristics of the stem cells, multipotency of the clonogenic cells was analyzed. Alizarin Red staining showed calcium deposition in the cells layer (Figure 1B). Safranin O staining was used to identify of chondrogenic pellet exhibited positive potential toward chondrogenic phenotype (Figure 1C). Newly differentiated adipocytes were shown through Oil Red staining of adipogenic cultures (Figure 1D). The mRNA levels of collagen type I, scleraxis, tenomodulin, and mohawk were higher in TDSCs compared with BMSCs and adipose tissue-derived stem cells (ADSCs) (Figure 1E).

### Effects of Tectorigenin on Rat TDSC Viability and Gene Expression *in vitro*

**FIGURE 2:**
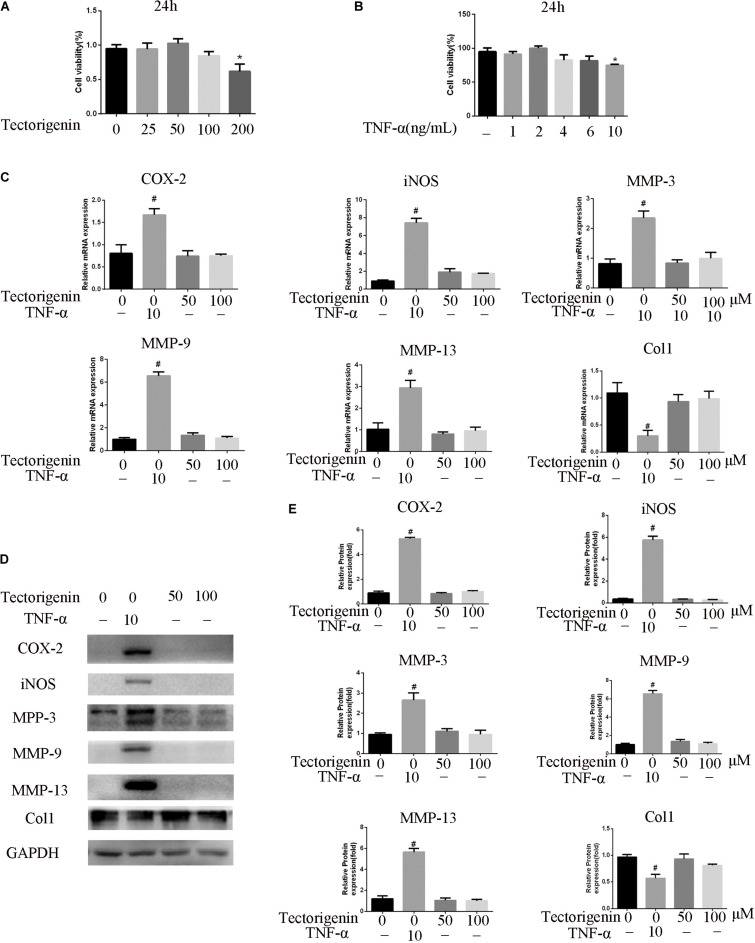
The effect of tectorigenin and TNF-α on TDSCs viability. **(A)** TDSCs were incubated with various concentrations of tectorigenin for 24 h, and CCK-8 was set to measure the role of tectorigenin on TDSC viability. **(B)** Several concentrations of TNF-α were added to TDSCs for 24 h, and CCK-8 was conducted to evaluate the effect of TNF-α on TDSC viability. **(C)** TDSCs were treated with tectorigenin (50 and 100 μM) for 1 h and then incubated with TNF-α (10 ng/ml) for 24 h. **(D,E)** The expression of inflammatory markers, MMPs, and Col1 in TDSCs treated with 0, 50, and 100 μM tectorigenin in the presence and the absence of TNF-α using qRT-PCR and western blotting. The mean ± standard deviation was used to express the data, *N* = 3. *^#^P* < 0.05 vs. control group and *P < 0.05 vs. TNF-α group. MMPs, matrix metalloproteinase; COX-2, cyclooxygenase-2; iNOS, inducible nitric synthase; TNF-α, tumor necrosis factor-α; Col1, collagen I.

As shown in Figure 2, the CCK-8 assay was used to evaluate the cytotoxicity of tectorigenin. Tectorigenin at concentrations of 0, 10, 25, 50, 100, and 200 μM were tested for 24 h, and the results showed that tectorigenin has no significant inhibition on TDSCs at concentrations ≤ 100 μM (Figure 2A). Thus, in this work, concentrations at 50 and 100 μM were used. Furthermore, tectorigenin did not have a significant impact on MMP-3, MMP-9, MMP-13, inducible nitric synthase (iNOS), cyclooxygenase-2 (COX-2), or collagen I in TDSCs at the protein and mRNA levels (Figures 2C,E,F).

### TNF-α Affects the Viability of TDSCs and Upregulates the Expression of MMPs and Inflammatory Genes *in vitro*

Similarly, CCK-8 was used to detect the influence of TNF-α on TDSCs. As shown in Figure 2B, TNF-α at concentration of 10 ng/ml showed notable suppression in TDSCs. Thus, in this experiment, 10 ng/ml was chosen. Furthermore, the effect of TNF-α at the 10-ng/ml concentration on MMP-3, MMP-9, MMP-13, iNOS, COX-2, and collagen I was examined at the mRNA and protein levels. The results showed that TNF-α is able to induce the expression of MMP-3, MMP-9, MMP-13, COX-2, and iNOS, as well as decreasing the expression of collagen I (Figures 2C,E,F).

### Tectorigenin Inhibits TNF-α-Induced TDSC Matrix-Degradation and Inflammatory Markers in Rat TDSCs and Alleviates Tendinopathic TDSCs

**FIGURE 3:**
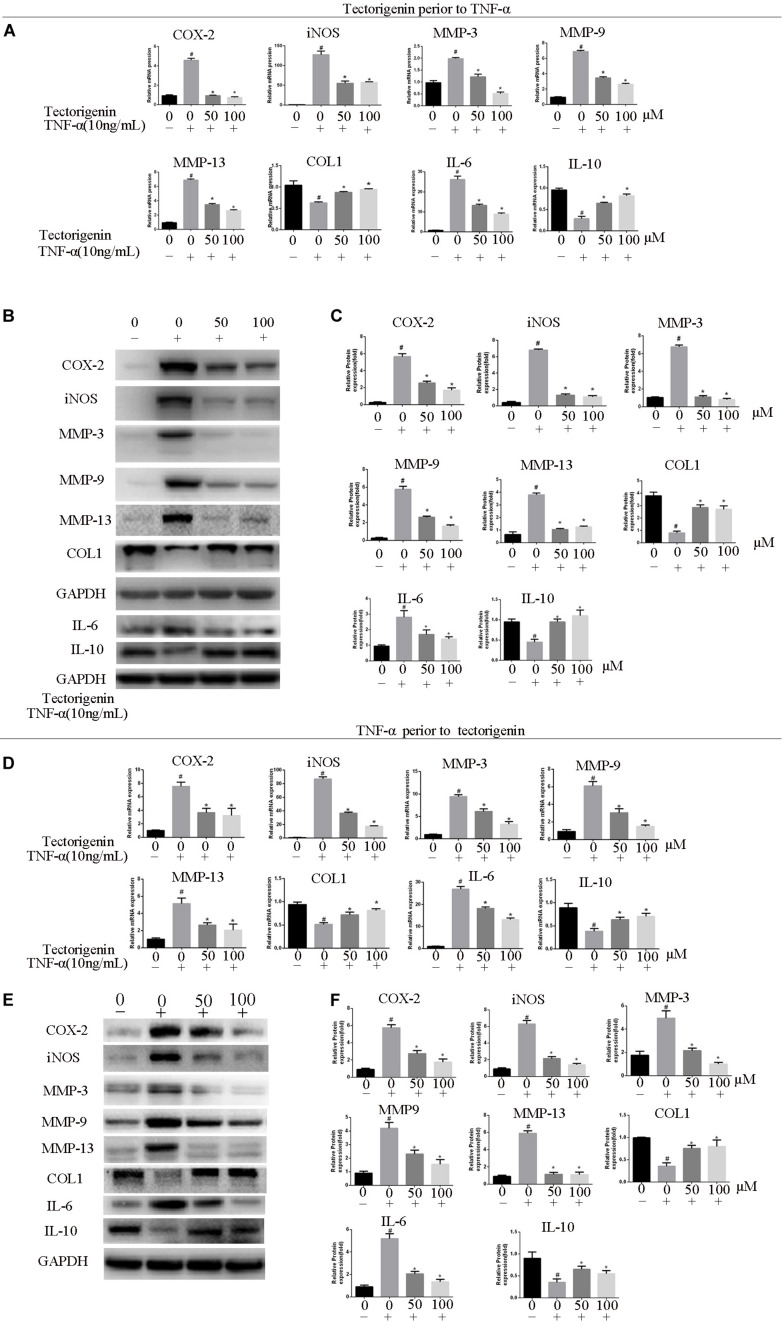
Tectorigenin inhibits high expression levels of COX-2, iNOS, IL-6, and MMPs and upregulates collagen type I and IL-10 in TNF-α-induced tendinopathy in TDSCs. TDSCs were pretreated with tectorigenin and then incubated with TNF-α (10 ng/ml) for 24 h. **(A)** The expression of inflammatory markers, MMPs, IL-10, and collagen I were evaluated at mRNA level using qRT-PCR. **(B,C)** Western blot was used to evaluate the expression of inflammatory markers, MMPs, IL-10, and collagen I at the protein level. TDSCs were stimulated with TNF-α to stimulate the inflammation for 12 h, and then incubated with tectorigenin for 24 h. **(D–F)** The mRNA and protein levels of the changes of MMP-3, MMP-9, MMP-13, COX-2, iNOS, IL-6, IL-10, and collagen I. The mean ± standard deviation was used to express the data, *N* = 3. *^#^P* < 0.05 vs. control group and *P < 0.05 vs. TNF-α group. MMPs, matrix metalloproteinase; COX-2, cyclooxygenase-2; iNOS, inducible nitric synthase; TNF-α, tumor necrosis factor-α; Col1, collagen I.

**FIGURE 4:**
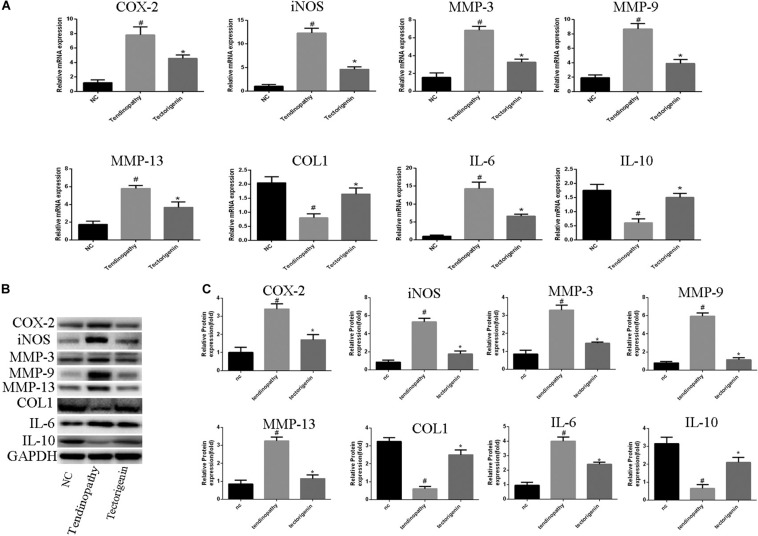
The role of tectorigenin in tendinopathic rat TDSCs. The rats were received full Achilles tendon transection, and then the cells were isolated and seeded. The medium was substituted every 2 days with or without 100 μM tectorigenin. **(A)** The expression of inflammatory markers, MMPs, IL-10, and collagen I were evaluated at mRNA level using qRT-PCR. **(B)** Western blot was used to evaluate the expression of inflammatory markers, MMPs, IL-10, and collagen I at the protein level. The mean ± standard deviation was used to express the data, *N* = 3. *^#^P* < 0.05 vs. control group and *P < 0.05 vs. tendinopathic cell group. MMPs, matrix metalloproteinase; COX-2, cyclooxygenase-2; iNOS, inducible nitric synthase; Col1, collagen I; IL-6, interleukin 6; IL-10, interleukin 10.

The effect of tectorigenin on TNF-α-induced TDSC matrix-degrading enzyme inflammation enzyme expression in rat TDSCs and in tendinopathic cells was evaluated. For normal rat TDSCs, the cells were treated with tectorigenin at concentrations 50 and 100 μM for 1 h, and then incubated with TNF-α (10 ng/ml) for 24 h. The mRNA expressions of MMP-3, MMP-9, MMP-13, iNOS, COX-2, IL-6, IL-10, and collagen I were quantified using q-RT PCR. As shown in Figure 3A, the mRNA levels of MMP-3, MMP-9, MMP-13, iNOS, COX-2, IL-6, IL-10, and collagen I were alleviated by tectorigenin treatment. For protein assessment, TDSCs were cultured and treated with tectorigenin for 1 h, and then incubated with TNF-α (10 ng/ml) for 24 h. The cells were harvested and western blot was conducted to evaluate the protein levels of MMP-3, MMP-9, MMP-13, COX-2, iNOS, IL-6, IL-10, and collagen I. As shown in Figures 3B,C, tectorigenin at concentrations of 50 and 100 μM is able to inhibit the TNF-α-induced high expression of MMP-3, MMP-9, MMP-13, COX-2, iNOS, and IL-6 and increase the expression of collagen I and IL-10. In addition, TDSCs were stimulated with TNF-α for 12 h to stimulate the inflammation, and then incubated with tectorigenin for 24 h. The mRNA and protein levels were measured to detect the changes of MMP-3, MMP-9, MMP-13, COX-2, iNOS, IL-6, IL-10, and collagen I. Interestingly, the results showed the ability of tectorigenin to reduce the activation of those markers (Figures 3D–F). For the cells which were harvested from tendinopathic rats, they were divided to tendinopathy and tectorigenin treatment groups. The markers which have been described above were also evaluated at the mRNA and protein levels (Figure 4).

### Tectorigenin Inhibits TNF-α-Induced TDSC Apoptosis

**FIGURE 5:**
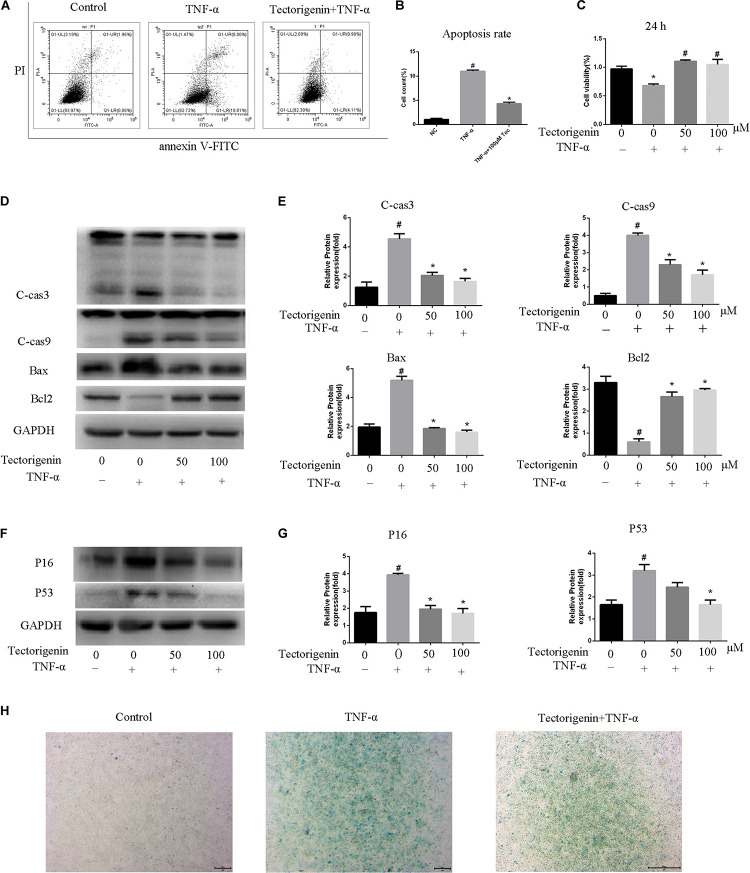
The effects of tectorigenin on TNF-α-induced apoptosis and senescence of TDSCs. TDSCs were pretreated with tectorigenin for 1 h and then incubated with TNF-α for 24 h. **(A,B)** Apoptosis was measured using annexin V-FITC/PI kit. **(C)** CCK-8 showed the effect of tectorigenin on TNF-α-induced suppression of TDSCs viability. **(D.E)** Western blot analysis of apoptosis markers, C-Cas 3, C-Cas9, Bax, and Bcl_2_. The mean ± standard deviation was used to express the data, *N* = 3. *^#^P* < 0.05 vs. control group and *P < 0.05 vs. TNF-α group. TNF-α, tumor necrosis factor-α; C-Cas, cleaved caspase-3; C-Cas 9, cleaved caspase-9. **(F,G)** Western blotting analysis of P16 and P53 at protein level. **(H)** β-Galactosidase activity assay in TDSCs. The mean ± standard deviation was used to express the data, *N* = 3. *^#^P* < 0.05 vs. control group and *P < 0.05 vs. TNF-α group. TNF-α, tumor necrosis factor-α; C-Cas, cleaved caspase-3; C-Cas 9, cleaved caspase-9.

The flow cytometry showed that tectorigenin is able to decrease TNF*-*α-induced apoptosis (Figure 5A). CCK-8 assay was performed to verify whether tectorigenin can protect TDSCs against TNF*-*α. As shown in Figure 5C, tectorigenin is able to suppress TNF*-*α-induced apoptosis in rat TDSCs. Western blotting analysis was used to evaluate the effect of tectorigenin against TNF*-*α-induced apoptosis, thus the protein levels of cleaved caspase-3, cleaved caspase-9, BAX, and Bcl_2_ were examined in this experiment. The findings reveal that tectorigenin has the ability to decrease the TNF*-*α-induced apoptosis in TDSCs (Figures 5D,E).

### Tectorigenin Alleviates TNF-α-Induced TDSC Senescence

The protein level expressions of P53 and P16 were evaluated by western blotting. The data showed that cells incubated with TNF*-*α reflected high expression levels of P53 and P16, while tectorigenin decreased them (Figures 5F,G). Furthermore, the cells treated with TNF*-*α expressed a high level of SA-β-Gal-positive TDSCs compared with the negative control group, and likewise tectorigenin was able to ameliorate this (Figure 5H).

### Tectorigenin Attenuates the Ossification in TDSCs

**FIGURE 6:**
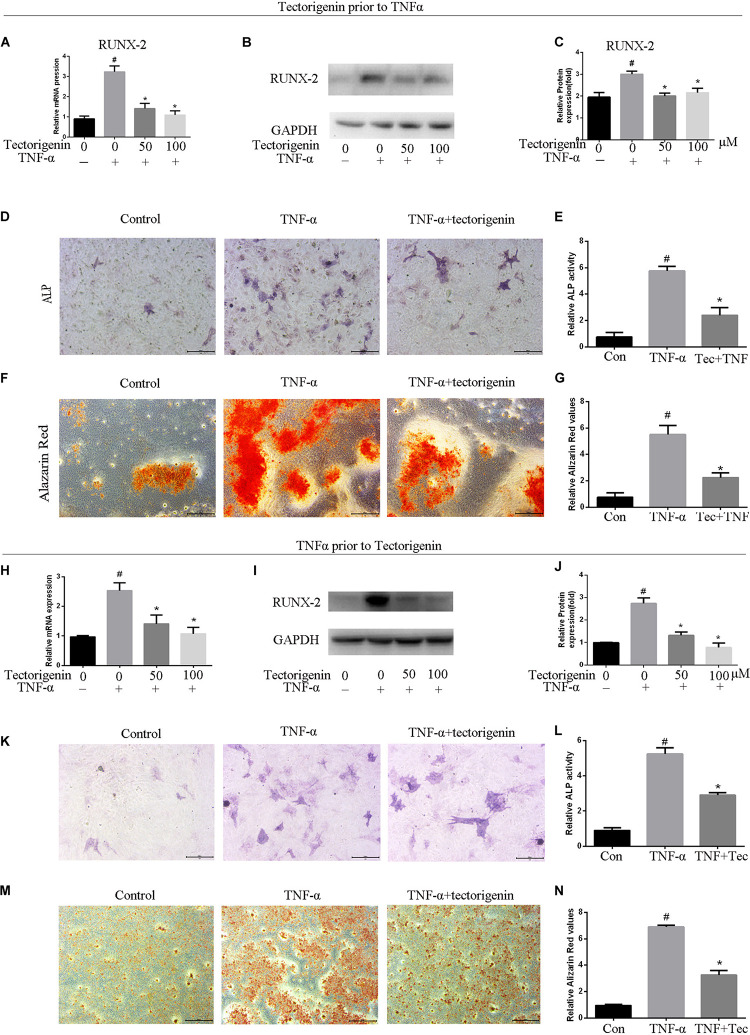
Tectorigenin alleviates ossification in TDSCs. The cells were cultured in osteogenic induction medium in companion with tectorigenin or TNF-α for 7 and 14 days. **(A)** mRNA level of RUNX-2 analyzed using qRT-PCR. **(B,C)** Protein level of RUNX-2 analyzed using western blot analysis. **(D,E)** ALP staining was set for 7 days and quantitative analysis, Scale bar = 200 μM. **(F,G)** Alizarin Red staining was conducted in TDSCs cultured for 14 days and quantitative analysis, Scale bar = 500 μm. The second part of this figure (TNF-α prior to tectorigenin), the cells were stimulated with TNF-α followed by tectorigenin treatment. **(H)** mRNA level of RUNX-2 analyzed using qRT-PCR. **(I,J)** Protein level of RUNX-2 analyzed using western blot analysis. **(K,L)** ALP staining was set for 7 days and quantitative analysis, Scale bar = 200 μM. **(M,N)** Alizarin Red staining was conducted in TDSCs cultured for 14 days and quantitative analysis, Scale bar = 500 μm The mean ± standard deviation was used to express the data, *N* = 3. *^#^P* < 0.05 vs. control group and *P < 0.05 vs. TNF-α group. TNF-α, tumor necrosis factor-α; ALP, alkaline phosphatase staining.

The mRNA and protein levels of RUNX-2 were evaluated using qRT-PCR and western blotting (Figures 6A–C). ALP staining and Alizarin Red staining were performed, and the results showed that TNF*-*α aggravated osteogenic differentiation in TDSCs. Treatment with tectorigenin showed alleviation of the ossification in TDSCs. As shown in Figure 6, RUNX-2 expression was higher in the TNF*-*α-treated group compared with the sham group and was reduced with treatment of tectorigenin. ALP staining and Alizarin Red staining showed that TNF*-*α induced osteogenic differentiation of TDSCs, while tectorigenin decreased these changes (Figures 6D–G). In this study, we stimulated the cells with TNF*-*α and then followed by tectorigenin treatment to measure the ability of tectorigenin to attenuate the ossification of TDSCs. The results showed that tectorigenin is able to alleviate TNF*-*α-induced ossification of TDSCs (Figures 6H–N).

### Tectorigenin Reduces TNF-α-Induced Activation of NF-κB and MAPK Signaling Pathway *in vitro*

**FIGURE 7:**
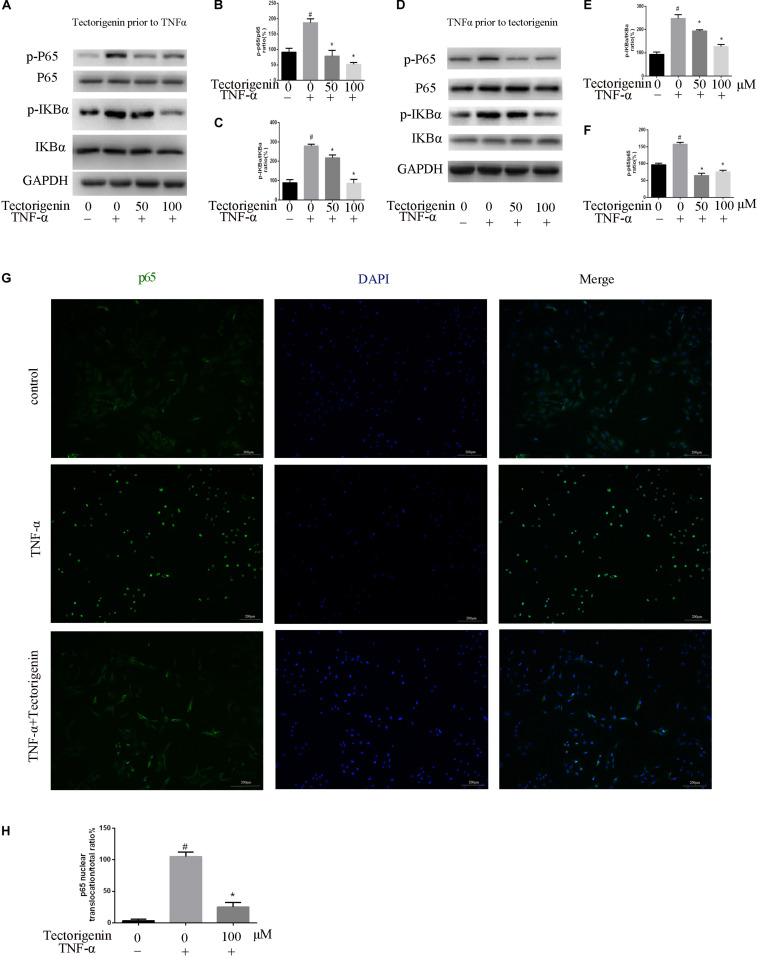
Tectorigenin decreased the TNF-α-induced activation of nuclear factor-kappa B in TDSCs. TDSCs were pretreated with tectorigenin for 1 h and then incubated with TNF-α for 30 min. **(A–C)** Western blot and quantitative analysis relevant to p-P65/P65 and p-IKBα/IKBα. **(D–F)** Western blotting and the quantitative analysis relevant to p-P65/P65 and p-IKBα/IKBα of cells pretreated with TNF-α for 30 min and then incubated with tectorigenin for 1 h. **(G,H)** The nuclear translocation of P65 was detected by immunofluorescence microscopy. Green, p65; blue, DAPI. Scale bar = 100 μM. The mean ± standard deviation was used to express the data, *N* = 3. *^#^P* < 0.05 vs. control group and *P < 0.05 vs. TNF-α group. TNF-α, tumor necrosis factor-α; IKBα, nuclear factor-kappa B inhibitor α.

**FIGURE 8:**
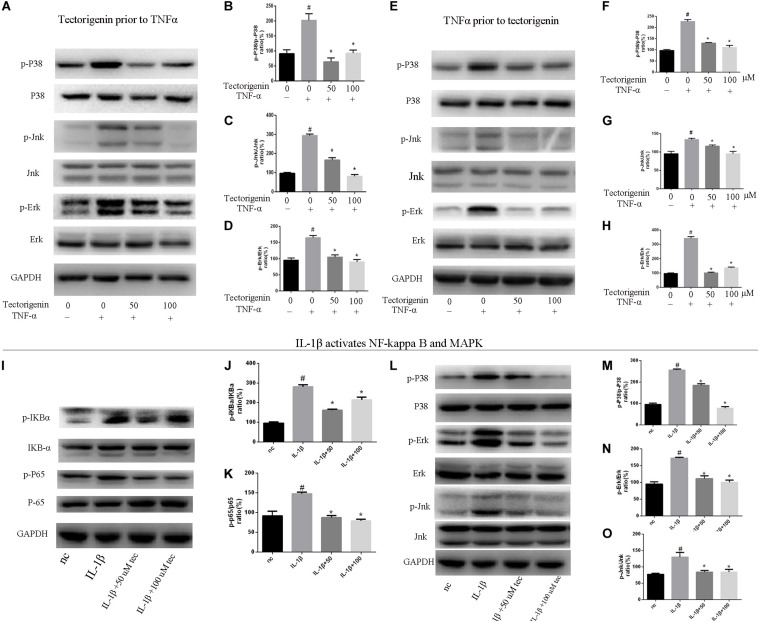
Tectorigenin decreased the TNF-α-induced activation of MAPK and IL-1β-induced the activation of MAPK and NF-κB pathways in TDSCs. TDSCs were pretreated with tectorigenin for 1 h and then incubated with TNF-α (10 ng/ml) for 30 min. **(A)** Western blot analysis and **(B–D)** relevant quantitative analysis for p-P38, P38, Jnk, p-Jnk, Erk, and p-Erk. **(E–I)** Western blot analysis and relevant quantitative analysis for p-P38, P38, Jnk, p-Jnk, Erk, and p-Erk of cells pretreated with TNF-α for 30 min and then incubated with tectorigenin for 1 h. TDSCs were pretreated with tectorigenin for 1 h and then incubated with IL-1β for 30 min. **(I–K)** Western blot and quantitative analysis relevant to p-P65/P65 and p-IKBα/IKBα. **(L)** Western blot analysis and **(M–O)** relevant quantitative analysis for p-P38, P38, Jnk, p-Jnk, Erk, and p-Erk. The mean ± standard deviation was used to express the data, *N* = 3. *^#^P* < 0.05 vs. control group and *P < 0.05 vs. TNF-α group. TNF-α, tumor necrosis factor-α; MAPK, mitogen-activated protein kinase; p-Erk, phosphor-Erk; p-Jnk, phosphor-Jnk; p-P38, phosphor-P38.

Tendon-derived stem cells were pretreated with tectorigenin at concentrations of 0, 50, and 100 μM for 1 h, and then stimulated with TNF-α at 10 ng/ml for 30 min. Western blot assay was used to measure the protein levels of NF-κB (p65 and IκBα) and MAPK (p38, Eer, and Jnk). As shown in Figures 7, 8, TNF-α activated NF-κB and MAPK pathways and upregulated the phosphorylation levels of p65, IκBα, P38, Erk, and Jnk. With tectorigenin treatment, all the TNF-α activations were inhibited. Furthermore, immunofluorescence showed that TNF-α-induced P65 translocation into nucleuses of TDSCs was blocked by treatment with 100 μM tectorigenin (Figure 7G). In addition, TDSCs were stimulated with TNF-α for 30 min, and then incubated with tectorigenin for 1 h. Western blotting was conducted to detect the changes of NF-κB and MAPK signaling pathways. Interestingly, the results showed the ability of tectorigenin to reduce the activation of NF-κB (Figures 7D–F) and MAPK (Figures 8E–I). These results indicate that tectorigenin is able to inhibit the TNF-α-induced activation of NF-κB and MAPK pathways in TDSCs. Furthermore, tectorigenin is able to reduce IL-1β-induced activation of NF-κB and MAPK signaling pathways. The cells were incubated with tectorigenin for 1 h and stimulated with IL-1β at 10 ng/ml for 30 min. The results showed that tectorigenin is able to inhibit IL-1β-induced activation of NF-κB and MAPK signaling pathways (Figure 5).

### Tectorigenin Alleviates Tendinopathy *in vivo* in Rat Model

**FIGURE 9:**
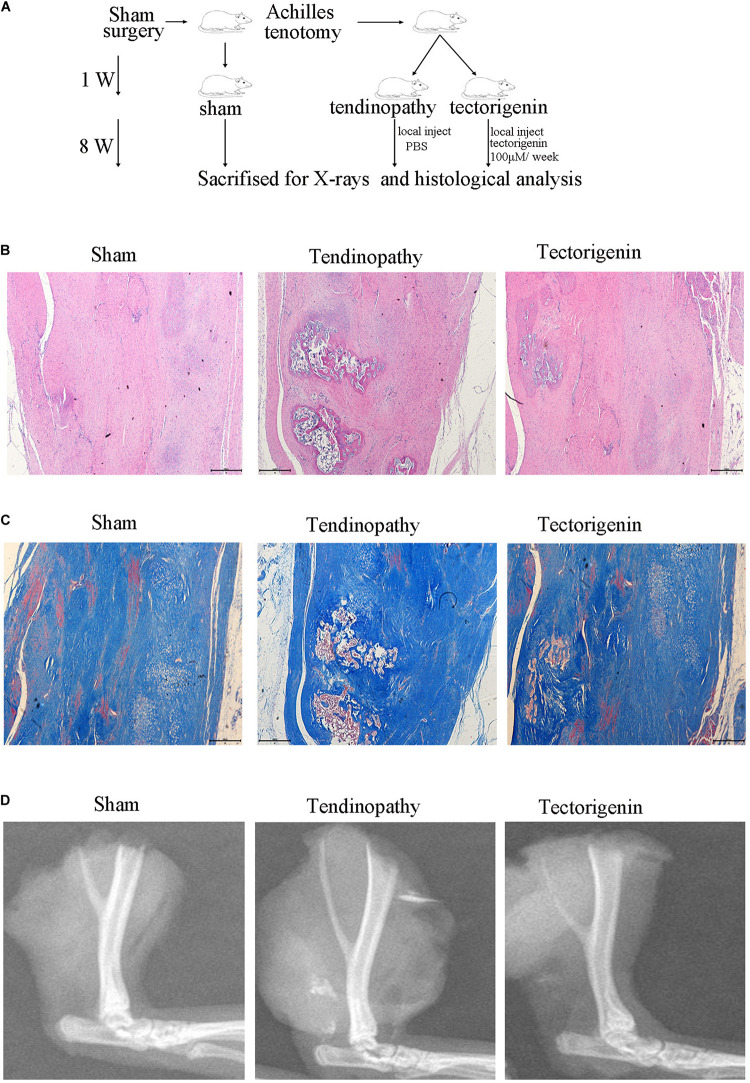
Tectorigenin alleviates calcification of tendon. **(A)** A schematic representation for the *in vivo* study. **(B)** HE staining of Achilles’s tendon for three groups, Scale bar = 500 μM. **(C)** Modified Masson staining of Achilles’s tendon for three groups, Scale bar = 500 μM. **(D)** X-ray images of Achilles’s tendon for three groups.

**FIGURE 10:**
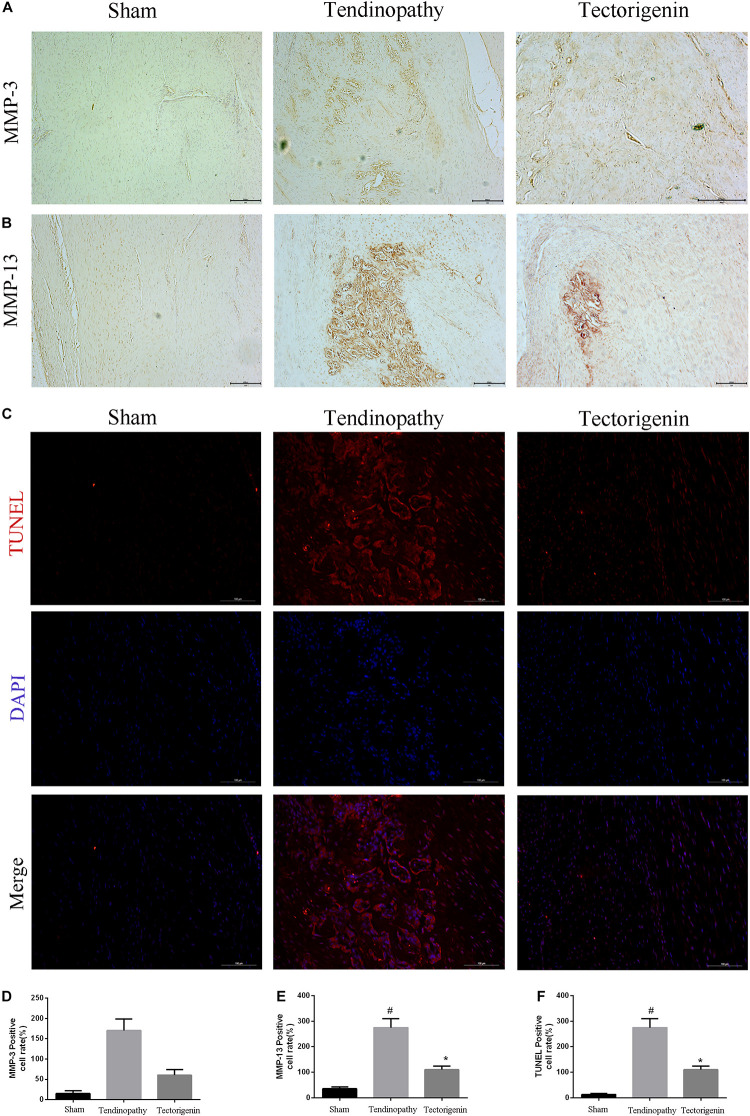
The protective effect of tectorigenin in a rat tendinopathy model. **(A,B,D,E)** Immunohistochemistry for antibodies against MMP-3 and MMP-13 and quantitative analysis. **(C,F)** TUNEL staining of Achilles’s tendon for three groups, Scale bar = 200 μM. ^#^*P* < 0.05 vs. control group and *P < 0.05 vs. Tendinopathy group.

To develop the tendinopathy model, a total of 12 rats received full Achilles tendon transection and were divided into tendinopathy model and tectorigenin-treated groups. Sham surgery was used for the six rats in the normal group. After 7 days of tendon transection, tectorigenin solution was injected weekly into the area between the Achilles tendon and skin in the tectorigenin-treated group for 8 weeks (Figure 9A). The effect of tectorigenin on rat tendinopathy *in vivo* was evaluated by HE staining and modified Masson staining. The arrangement of collagen fibers and fibroblasts was disrupted in the tendinopathy group compared with the sham group, and tectorigenin was able to reverse this (Figures 9B,C). Furthermore, as shown on X-ray image, tectorigenin was able to reverse calcification in Achilles tendons (Figure 9D). Additionally, immunohistochemistry staining confirmed that the tectorigenin-treated group had a lower expression of MMP-3 and MMP-13 compared with the positive control groups (Figures 10A,B,D,E). TUNEL staining was set to assess the apoptosis of tendon fibroblasts and showed that administration of tectorigenin can protect cells against apoptosis (Figures 10C,F). Collectively, these findings indicate that tectorigenin is able to alleviate tendinopathy in the *in vivo* rat model.

## Discussion

Tendon-derived stem cells are able to differentiate into osteoblasts, tenocytes, adipocytess, chondrocytes, and fibroblasts (Lui, 2015). Among these cells, TDSCs tend to differentiate toward tenocytes (Bi et al., 2007). TDSCs serve a vital role in the healing process of tendon after injury; however, the failure to heal may lead to tendon ossification (Yee Lui et al., 2011; Chaudhury, 2012). Thus, in this study, we investigate the effect of tectorigenin on tendinopathy in TDSCs *in vitro*.

TNF-α is well-known to inhibit the proliferation of TDSCs (Frasca et al., 2012) and induce apoptosis of TDSCs (Han et al., 2017). TNF-α can upregulate proinflammatory markers such as MMPs and IL-1 (Feldmann and Maini, 2001). Previous studies have demonstrated the association between chronic tendinopathy and the upregulation of TNF-α (Rath and Aggarwal, 1999).

In the present study, we confirmed that TNF-α induces TDSC inflammation and apoptosis as well as activates NF-κB and MAPK pathways in TDSCs. Furthermore, we verified that tectorigenin is able to reverse the TNF-α-induced dysfunction of TDSCs by regulating the NF-κB and MAPK pathways.

After the isolation of TDSCs and detection of stem cell markers, cell viability was evaluated. CCK-8 assay was used to analyze the effect of TNF-α and tectorigenin on TDSC viability. The results suggest that TNF-α suppresses TDSC viability at concentration of 10 ng/ml and tectorigenin at concentration of 200 μM. Thus, we conducted our study using 10 ng/ml TNF-α and 50 and 100 μM tectorigenin. Furthermore, we evaluated the role in tectorigenin in the absence of TNF-α on MMPs, collagen I, and inflammatory markers at both mRNA and protein levels. The results showed that tectorigenin has no obvious effects on the MMPs, collagen I, or inflammatory markers compared with the sham group.

It is widely accepted that the degenerative process of inflammation may lead to tendinopathy. Several inflammatory mediators, such as MMPs and COX-2 serve a role on the development of tendinopathy (Rees et al., 2014). MMPs are responsible for ECM remodeling and synthesis during tendon healing (Riley et al., 2002). COX-2 and iNOS have been known to participate in the progression of inflammatory diseases, and the suppression of iNOS and COX-2 has shown potential outcomes (Moita et al., 2013). Interleukin 6 (IL-6) was reported to inhibit the tenogenic markers of Achilles TDSC, such as collagen type I, scleraxis, and tenomodulin (Chen et al., 2018). In the present study, we found that TNF-α increased the expression of MMPs (MMP-3, MMP-9, and MMP-13), COX-2, IL-6, and iNOS, which were decreased in the tectorigenin treatment groups at both mRNA and protein levels. Previous studies showed that the increasing production of collagen I can enhance tendon healing (Chen et al., 2014). Interleukin (IL-10) is an anti-inflammatory cytokine that can block NF-κB pathway, and regulating of IL-10 family is promising for the treatment of human diseases (Wang et al., 2019b). Thus, we evaluated type I collagen and IL-10 at both mRNA and protein levels. The results showed that TNF-α decreased the level of collagen I and IL-10, while tectorigenin increased them.

Apoptosis is programmed cell death, which serves a vital role in tissue homeostasis. Apoptosis initiates several human diseases, such as autoimmune diseases and degenerative skeletomuscular diseases (Elmore, 2007). Generally, caspases, such as caspase-3, are tightly associated with apoptosis and are executioners of apoptosis. The regulatory proteins of apoptosis determine the fate of a cell; for example, Bax accelerates apoptosis, while Bcl-2 inhibits apoptosis (Oltvai et al., 1993). In the current study, TNF-α suppressed TDSC viability, upregulated apoptosis proteins (C-Cas3, C-cas9, Bax), and decreased the expression of Bcl-2, while it was alleviated by tectorigenin. Furthermore, TUNEL assay demonstrated that tendon ossification scars had a huge number of apoptotic cells in the tendinopathy group, which was alleviated by tectorigenin treatment. These outcomes suggest that tectorigenin exerts anti-apoptotic effects.

Additionally, the results showed that TNF-α can induce the senescence of TDSCs as well as increase the expression of P53 and P16. Senescence is an unrepairable limiting of cell growth and declination of self-renewal ability in stem cells. It is described by the increasing activity of SA-β-gal and high expression of P53 and P16 (Helman et al., 2016; Huang et al., 2017). Our results showed that tectorigenin is able to reduce the TNF-α-induced high expression of P53 and P16. Furthermore, tectorigenin decreased SA-β-gal-positive TDSCs.

Heterotopic ossification in tendons leads to joint mobility restriction and pain. The pathogenetic process of heterotopic ossification is associated with osteoprogenitor stem cells (TDSCs) in tendon tissues. The heterotopic ossification can be a result of osteoid formation, which can be initiated by the abnormal ossification of TDSC (Jiang et al., 2018). In this study, the outcomes showed that tectorigenin reduced the TNF-α-induced expression of RUNX-2 and decreased the osteogenic differentiation of TDSCs, made observable by ALP staining and Alizarin Red staining. *In vivo*, HE, and modified Masson staining showed that tectorigenin alleviated the ossification of tendon tissues.

A number of evidences have shown that MAPK and NF-κB pathways are involved in inflammation, apoptosis, and ossification; for example, ERK regulates apoptosis, while P38 mediates apoptosis and inflammation (Zhou et al., 2015). The activation of MAPK/Jnk pathway can enhance mineralization and increase ALP activity (Wang et al., 2014). The activation of NF-κB has been shown to induce the activation of MAPK pathway (Wang et al., 2019a). Our results showed that TNF-α initiated the activation of MAPK and NF-κB pathways. Tectorigenin blocked the phosphorylation of p65 and reduced the translocation of phospho-p65 from cytoplasm to nucleus. Furthermore, tectorigenin reduced the phosphorylation of NF-κB inhibitor α. At the same level, tectorigenin reduced the phosphorylation of p38, Jnk, and Erk. In this study, TDSCs were stimulated with another inflammatory markers (IL-1β) to evaluate that tectorigenin usage has interfered with MAPK and NF-κB pathways not directly interacting with TNF-α itself. The results showed the ability of tectorigenin to inhibit the activation of MAPK and NF-κB pathways.

## Conclusion

Our study showed that TNF-α insults TDSCs and contributes to the development of tendinopathy *via* increasing the activation of MAPK and NF-κB pathways. Treatment with tectorigenin reduced the activation of MAPK and NF-κB pathways in TNF-α-treated TDSCs, and thus played anti-inflammatory, anti-apoptotic, anti-senescence, and anti-ossification roles. Collectively, our outcomes have revealed a new agent for the treatment of tendinopathy.

## Data Availability Statement

The raw data supporting the conclusions of this article will be made available by the authors, without undue reservation.

## Ethics Statement

The animal study was reviewed and approved by all the animal experiment study was controlled and approved by the protocol Ethics Committee of the Second Affiliated Hospital, School of medicine, Zhejiang University, Hangzhou, China.

## Author Contributions

LW, YX, and SM took part in the designing of the experiments, contributed reagents, materials, and analysis tools. SM, KX, and ZW ran the experiments. SM, YH, LX, JR, and CM wrote the manuscript. SM and ZC participated in the analyzing of the data. All authors read and approved the final manuscript and listed have made substantial contributions to the study.

## Conflict of Interest

The authors declare that the research was conducted in the absence of any commercial or financial relationships that could be construed as a potential conflict of interest.
