# Supplementary figures and images for: Tectorigenin Alleviates Inflammation, Apoptosis, and Ossification in Rat Tendon-Derived Stem Cells via Modulating NF-Kappa B and MAPK Pathways
Source: Front Cell Dev Biol. 2020 Oct 22;8:568894. doi: 10.3389/fcell.2020.568894 (PMC7642480; doi:10.3389/fcell.2020.568894)

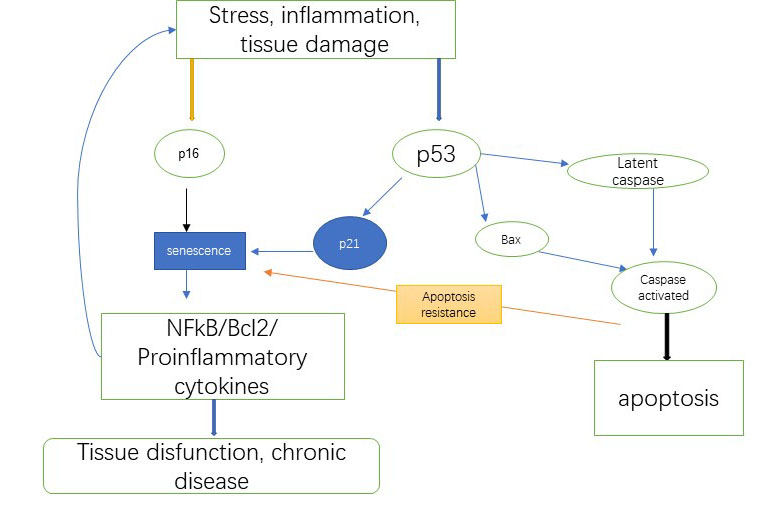

Supplement: Supplementary file 1 [file Image_1.JPEG]

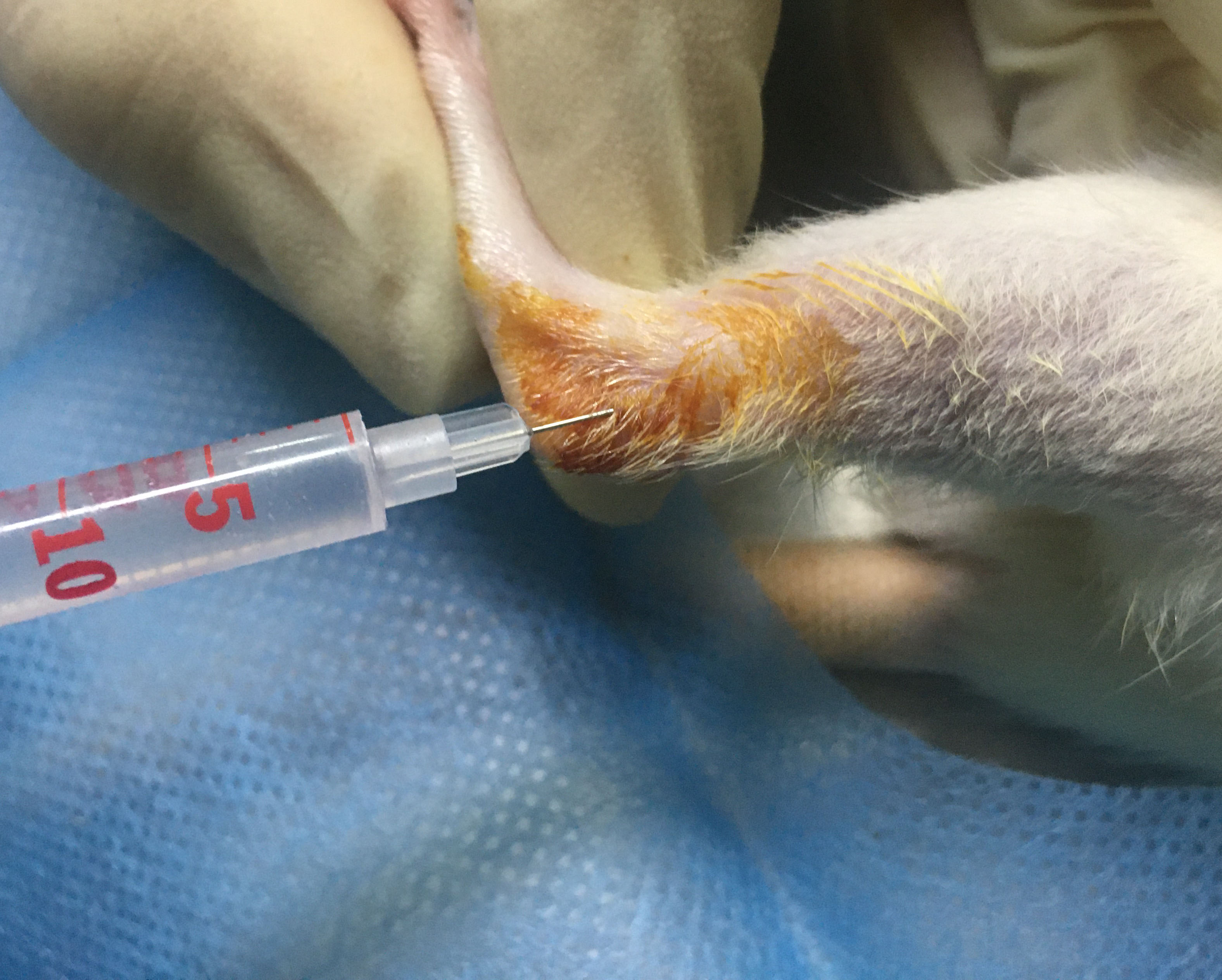

Supplement: Supplementary file 2 [file Image_2.JPEG]
